# Binding and Docking Interactions of NO, CO and O_2_ in Heme Proteins as Probed by Density Functional Theory

**DOI:** 10.3390/ijms10094137

**Published:** 2009-09-22

**Authors:** Vangelis Daskalakis, Constantinos Varotsis

**Affiliations:** 1 Institute of Electronic Structure and Laser, Foundation for Research and Technology - Hellas, P.O. Box 1527,GR-711 10 Heraklion, Greece; E-Mail:Chem487@edu.uoc.gr (V.D.); 2 Department of Chemistry, University of Crete, P.O. Box 2208, 71003, Voutes – Heraklion, Greece

**Keywords:** density functional theory, ligand molecules, protein cavities, proximal effect

## Abstract

Dynamics and reactivity in heme proteins include direct and indirect interactions of the ligands/substrates like CO, NO and O_2_ with the environment. Direct electrostatic interactions result from amino acid side chains in the inner cavities and/or metal coordination in the active site, whereas indirect interactions result by ligands in the same coordination sphere. Interactions play a crucial role in stabilizing transition states in catalysis or altering ligation chemistry. We have probed, by Density Functional Theory (DFT), the perturbation degree in the stretching vibrational frequencies of CO, NO and O_2_ molecules in the presence of electrostatic interactions or hydrogen bonds, under conditions simulating the inner cavities. Moreover, we have studied the vibrational characteristics of the heme bound form of the CO and NO ligands by altering the chemistry of the proximal to the heme ligand. CO, NO and O_2_ molecules are highly polarizable exerting vibrational shifts up to 80, 200 and 120 cm^−1^, respectively, compared to the non-interacting ligand. The importance of Density Functional Theory (DFT) methodology in the investigation of the heme-ligand-protein interactions is also addressed.

## 1. Introduction

We can divide NO, CO and O_2_ ligand molecules interactions inside a protein into two different groups. The first one, called the inner cavity interactions, results from interactions of the ligand molecules with amino acid residues inside the protein cavities and represents those ligand molecules in the unbound-to-the-metal states. The second one, called the bound interactions, deals with the bound forms of the ligand molecules and either the related changes in the coordination sphere of the associated metal or other interactions that influence their binding energies and chemistry. As for the latter we will focus on the proximal interactions in the heme bound forms of the ligand molecules and in the deprotonation/protonation events in the coordination sphere of the Cu_B_ metal of Cytochrome *c* Oxidase (CcO). In this paper we report previous theoretical studies from our lab, as well as new calculations on model systems dealing with both kinds of interactions, which taken together, we use the term “protein effect”. Several theoretical studies [1–11] primarily based on Density Functional Theory (DFT), have provided insight into the geometries, electronic structures, and binding energies for the NO, CO and O-O complexes.

### 1.1. Inner Cavity Interactions

Inner protein cavities play a crucial role in controlling the dynamics, as well as their reactivity in reactions with small ligands like O_2_, CO and NO by accommodation and/or docking [12–14]. These cavities act like local storage sites for small molecules near the active site, resulting in an increased effective concentration of the ligand. Neutral, positively or negatively charged residual side chains form such structural characteristics in the proteins. Thus, ligands interact with charged side chains or hydrogen bonding networks. In addition, they exert active role in the ligand binding process. Electrostatic interactions exert significant contribution in stabilizing the three dimensional architecture of either individual proteins or even between different biological systems. In addition, such interactions in or near the active site determine the reactivity and functionality of enzymatic systems. Thus, it is crucial for such events to be probed by theoretical calculations and compared to experimental data.

Myoglobin (Mb) is found in large concentrations in muscles storing and delivering molecular oxygen to mitochondria of red muscle cells. Theoretical characterization of CO/NO and O_2_ ligand binding to ferrous (Fe^2+^) Mb becomes a significant step towards a better understanding of more complex biological systems, like CcO or nitric oxide reductases (NOR). CO dissociates from the heme-iron of the active site, during photolysis of complexes like Mb-CO [15–17] or CcO *ba_3_*-CO [18] and transiently moves to a cavity nearby. Dynamics of such migration has been studied experimentally by ps and fs time-resolved (TR) [15,16], as well as infrared (FTIR) spectroscopy in conjunction with thermal desorption spectroscopy (TDS) [17]. Theoretical Simulations (like molecular dynamics) have probed the cavities near the active site to study ligand migration after photodissociation, and possible ligand pathways to and from the active site cavity [12,19–25]. Three different CO bound states to heme-iron (A_0_ 1,967 cm^−1^, A_1_ 1,945 cm^−1^, A_2_ 1,932 cm^−1^) have been identified, as well as a number of unbound/free ligand states (B_1_ 2,130 cm^−1^/B_2_ 2,120 cm^−1^, C and D) [15,17,26–29]. These differences in ν(CO) vibrational frequencies correspond to diverse interactions with the surrounding residues. ν(CO) in CcO *ba_3_* exerts vibrational frequencies at 2,131 and 2,146 cm^−1^ attributed to B_1_ and B_0_ respectively, [18] after *photodissociation* of the *ba_3_*-CO complex.

Heme-protein HemeAT [30] functions as a signal transducer and molecular O_2_ sensor. Three states of CO have been spectroscopically identified. Two of them are bound states, at 1,967 (no interactions of the CO with the environment) and 1,928 cm^−1^ (hydrogen bonding interactions with the bound CO). A third ν(CO) vibration at 2,065 cm^−1^ does not shift under photolytic conditions and has been attributed to free CO in a cavity [31].

By changing the electrostatic environment near the free NO, CO and O_2_ on small models and in b3lyp/6–31g(d, p) level of theory we calculate the stretching vibrational frequencies ν(NO), ν(CO), ν(^3^O_2_) to construct diagrams of frequencies vs. interactions. It is interesting to note that especially for the free, unbound CO molecule, ν(CO) can become as low as that of the bound CO, depending on the electrostatic interactions with amino acid side chains [31].

Aside from the importance of CO as a molecular probe, several biological processes involve NO, as a cellular molecular messenger that controls a number of physiological events. It is known to inhibit O_2_ reduction by CcO or to be involved in additional oxidation and nitrification degrading proteins [32,33] and DNA [34].

### 1.2. The Bound Interactions

NO, CO and O_2_ forms bound to heme groups are subject to distal or proximal interactions. Blomberg *et al.* [1] have shown that Cu_B_ in CcO has a similar effect on the binding energies of NO, CO and O_2_ as the distal histidine in Mb. The heme iron is coordinated to a proximal histidine (His93) making the iron five coordinated with a free binding site for dioxygen. Above the free binding site resides the so called distal histidine (His64), which can form a hydrogen bond to the sixth iron ligand. One of the key features of the distal histidine is the discrimination between NO, CO and O_2_, favoring O_2_ by mainly electrostatic interactions [28].

Hydrogen bonding networks that affect the basicity of an axial to the heme iron ligand to support oxidation states greater than Fe(III) have been reported [35,36]. CcO, sulfite reductase and CooA exhibit such proposed hydrogen bonding networks [35–38]. In the case of CcO, hydrogen bonding to the N_δ_-H hydrogen of proximal histidine is the crucial step in understanding how reactivity of the heme-iron is controlled by the proximal environment in heme proteins [39].

A number of studies have addressed various aspects of hydrogen bonding effects to the proximal side. Franzen [40] has studied the effect of hydrogen bonding to the proximal histidine by donors such as H_2_O, CH_3_COO- and CH_3_CONHCH_3_ on the ν(Fe-C) and ν(C-O) frequencies of CO bound models of peroxidases active site containing the catalytic Asp-His-Fe triad. Based on the DFT calculations, it was proposed that increased hydrogen bonding to the N_δ_-H proton causes a rise in negative charge density on the imidazole ring and alters significantly the donor character of the imidazole π-system leading, in consequence, to an increased σ-bonding by the N_ε_ lone pair. The charge density on the iron metal is moved into the π* orbitals of bound CO. Moreover, in another case, Hu *et al.* [41] studied several five-coordinate [Fe(II)(Por)(2-MeHIm)] derivatives by Mössbauer spectroscopy, showing that imidazole deprotonation leads to distinctly different axial and equatorial bond distances, also observed were significant deviations in the displacement of iron atom from the heme plane compared to neutral imidazole species. It was thus proposed that such a switch altering heme chemistry is important for the biological function of heme proteins in cases of reversible binding of O_2_.

Turning to CcO, the ν(CO) frequency of the transient Cu_B_-CO complex determines in great amount the conformational changes (*e.g.,* ligand dissociation) of the copper metal site in CcO, whereas both ν(CO) and ν(Cu-C) are reliable criteria for protonation/deprotonation phenomena. Therefore, we can derive structural information for the Cu_B_ His ligands from the ν(CO) frequency of the Cu_B_^1+^-CO complex. The negative slope in the diagram of the DFT-optimized distances, *d*(C-O) versus *d*(Cu-C), implies the presence of a π-back-bonding in the Cu_B_-CO complexes [42].

In the rest of the paper, we will focus on new theoretical models probing such as the above mentioned interactions and discussion in general to include previous theoretical works and conclusions. The accuracy of the B3LYP functional in DFT methodology has been tested in the extended G3 benchmark set [43], which consists of enthalpies of formation, ionization potentials, electron affinities and proton affinities for molecules containing first- and second-row atoms. The B3LYP functional gives an average error of 4.3 kcal/mol [43] for 376 different molecules.

## 2. Results and Discussion

Results will be focused, initially, on the unbound forms of the CO, NO and O_2_ ligands (Section 2.1) and then theoretical calculations will be reported for metal-ligand complexes, such as Cu_B_-CO from CcO, heme-Fe-NO and heme-Fe-CO (Section 2.2).

### 2.1. The Unbound Interactions inside Cavities

**Figure 1.**
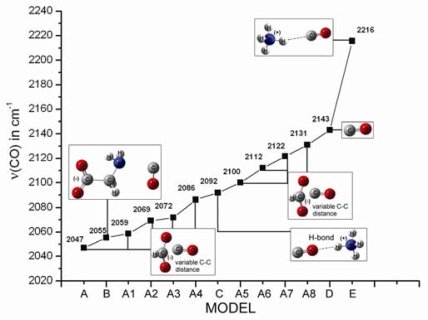
Empirical diagram depicting the polarizability extend, expressed in wavenumbers, of CO molecule inside protein cavities (figure taken from *Proc. Natl. Acad. Sci. USA* **2006**, *103*, 14796–14801 [31]).

Recently [31], we have applied density functional theory to investigate the effect of charged residues on ν(CO) of a docked CO molecule in a protein cavity. Because of the nature of these cavities, theoretical calculations have to be performed on simplified models with a restricted number of atoms. In these models, the charged residues in the protein cavity are represented by NH_4_^+^ (*e.g.,* the arginine side chain) and HCOO- (*e.g.,* aspartic acid). Figure 1 shows the models in which the free CO interacts with (i) HCOO- (model A, HCOO-…CO), (ii) a simple molecule that provides both carboxyl and amino groups, such as Gly (model B), and (iii) NH_4_^+^ (model C: CO…NH_4_^+^, model E: NH_4_^+^…CO). In the case of two NH_4_^+^ molecules (model not shown), two unconstrained NH_4_^+^ molecules strongly repel each other, whereas a constrained model (with fixed NH_4_+…CO…NH_4_+ distances) exhibits a large positive binding energy. Models A1–A8 show the effect of the C–C distance (negative charge near CO) on ν(CO). In model A, the C–C distance is optimized at 2.98 Å without using constraints, whereas models A1–A8 have constrained C–C distances at 3.08, 3.18, 3.58, 3.78, 3.98, 4.18, 4.38, and 4.58 Å, respectively. All models except for A1–A8 are optimized without constraints. In model B, in which both the amino and carboxyl groups interact with the CO, the carboxyl group is deprotonated, whereas the amino group is neutral. Model C contains hydrogen-bonding interaction in addition to the positive charge effect on CO.

Figure 1 shows the calculated ν(CO) trend versus the model. Positive charges around CO increase ν(CO), whereas negative charges decrease it. Straub and Karplus [44] have calculated the ν(CO) shifts in different CO–Imidazole dimmer complexes at the 4–21G basis set level to vary between −17 cm^−1^ (H bonding C–O…H–N) to +36 cm^−1^ (N–H…C–O). Moreover, Nutt and Meuwly [45] have implemented molecular dynamics simulations on the photodissociated state of carboxymyoglobin based on a three-site charge model for CO to calculate the IR spectra of the free CO molecule in the heme pocket [45]. The IR spectrum obtained exhibits peaks between 2170 and 2200 cm^−1^, corresponding to the signal from a single CO molecule docked in Mb. These signals are sensitive to the precise position and orientation of the CO molecule, as well as to the effect of the environment of the protein matrix (Mb) on the CO molecule. In general, the stretching frequencies ν(CO) of heme-bound and photodissociated CO serve as powerful tools to probe the electrostatic fields and accessible space in the vicinity of the CO molecule inside the protein matrix. The docked, photodissociated CO ligands in the protein matrix display IR peaks, providing strong evidence for the existence of structurally well defined docking site(s). In analogy to the bound CO forms, the ν(CO) of the docked CO is affected by the local environment through Stark effects of the local electric field acting on the CO dipole [12]. As our model tends to be more general and represent a wide variety of protein cavities, we have constructed an empirical diagram of ν(CO) to investigate the effect of positive or negative charges and that of H-bonding interactions to different orientations of CO. The empirical diagram shows a significant variability in ν(CO) ranging from 2047 cm^−1^ (interaction with only a negatively charged COO- group) to 2216 cm^−1^ (interaction with only a positive charge). The calculations also show that ν(CO) appears in the range of 2047–2131 cm^−1^ at 10 cm^−1^ steps when the CO distance to a negatively charged carboxyl group is varied. The combined interaction of carboxyl and amino groups to CO has a strong effect on ν(CO), as shown by the 88 cm^−1^ down-shift from 2143 cm^−1^ of gas CO, even though CO is not interacting directly with the deprotonated carboxyl.

**Figure 2.**
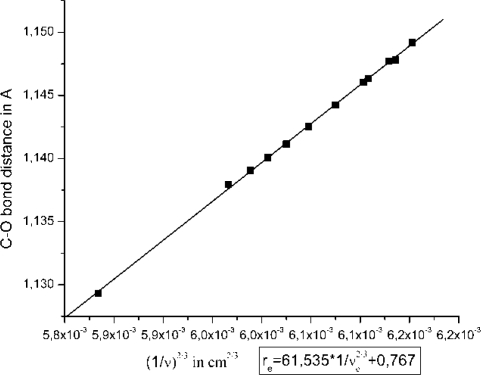
Parameter fitting for Badger’s rule on CO molecule based on the DFT models (figure taken from *Proc. Natl. Acad. Sci. USA* **2006**, *103*, 14796–14801, *S.I.,* [31]).

**Figure 3.**
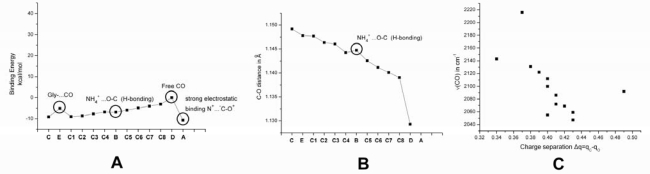
(A) Binding energies of CO models. (B) C-O distances in Å of CO models. (C) Charge separation Δq = q(C) – q(O) observed in the CO models.

All above calculated vibrational frequencies are consistent with the Badger’s rule [46]: ***r_e_*** = c_ij_(1/ν_e_^2/3^) + d_ij_, where *r_e_* is the equilibrium C–O distance and ν_e_ refers to ν(CO) (Figure 2). The empirical parameters c_ij_ and d_ij_ were calculated to be 61.535 and 0.767, respectively. We observe a huge diversity in ν(CO), reaching values in the region of CO bound metal complexes, like Cu_B_-CO in CcO [18]. In Figure 3 we draw three diagrams concerning trends for ν(CO)/CO: **(A)** binding energies for all models, **(B)** C–O distances in *angstroms* and **(C)** ν(C–O) frequencies in relation to Δq = q_C_–q_O_ charge separation (charge differemce between calculated carbon q_C_ and oxygen q_O_ charges) in the different A, A1–8, B, C, D and E cases.

The CO molecule can exist in three different resonance forms, depending on the environment or metal ligation. The different resonance structures of CO are linked through the below equilibrium (experimental interatomic distances are also shown for clarity):

$$:\overset{\left(-\right)}{C}\equiv \overset{\left(+\right)}{O}:(1.11\overset{\circ }{\text{A}})↔\;:C=O_{-}^{-}(1.23\overset{\circ }{\text{A}})↔\;:\overset{\left(+\right)}{C}-\overset{\left(-\right)}{O_{-}^{-}}:(1.43\overset{\circ }{\text{A}}).$$

When a positively charged atom is near carbon atom of CO, the first form is the dominant one, as in that case, electrostatics (attraction) are the main cause of stabilizing such interaction. Further polarization of CO molecule towards this direction would lead to a further strengthening of the C-O bond (model **E**). In the opposite trend, if a negatively charged molecule approaches carbon atom of CO, the last structure would prove to be the dominant one, as the C-O bond length is increased, while its strength is in turn decreased (models **A, A1–8**). A positive charge on the site of oxygen of CO would also lead to the third resonance structure, while an hydrogen bond would strengthen the C-O bond. In fact, these opposite effects collaborate in model **C** which includes positive charge and the hydrogen bonding interaction (CO…NH_4_^+^), as ν(CO) appears at 2,092 cm^−1^ taking into account both contributions. In the case of Gly-CO interaction, as in model **B**, the C-O bond weakens and the third resonance structure dominates (C-O distance at 1.43 Å). This could be due to a more complicated interaction between Gly and CO including a combination of electrostatic interactions from NH_2_, as well as COO-. Binding energies in Figure 3 do not exert a smooth trend versus the d(C-O) distances. This is due to a) hydrogen bonding involved in CO interactions (model **C**) and b) in the intense electrostatic interaction in model **E**. Such trends are also depicted in the ν(CO) versus Δq = q_C_–q_O_ charge separation diagram in the same figure. The negative slope indicates that as the charge separation between C and O increases (due to polarization), the C-O bond energy is decreased. Thus, calculations are fully consistent to the above mentioned equilibrium of CO resonance states.

In the models with NH_4_^+^ interacting with either O_2_ or NO we introduce, in addition to the positive charge effect, a hydrogen bond, not always present in the CO models with NH_4_^+^. Electrostatic fields, present in the protein cavities (or DFT models presented in this study) are responsible for a Stark Effect. An electric field induces a shift in ν(CO) in addition to a change in dipole moment, μ, of CO due to excitation by absorption of a vibrational mode. DFT studies by Brewer and Franzen [47] indicate that for carbonyls and NO moieties, transitions in molecular geometry, due to an electric field, are the main contributors to a Stark Effect.

**Figure 4.**
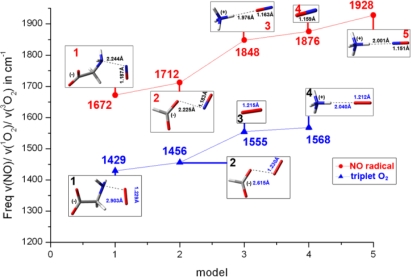
Empirical diagram depicting the extent of polarizability, expressed in wavenumbers, of NO and triplet O_2_ molecules inside protein cavities.

Moreover, we simulate intercavity interactions inside a protein in cases where the ligand molecule is different from CO, by replacing it by either NO, or ^3^O_2_ in the above presented models. Again, free ligands interact with charged or neutral moieties acting as amino acid side chains. Results are shown on Figure 4. All NO, CO and O_2_ ligand-gases seem to behave in a similar way exhibiting a pronounced diversity in vibrational stretching frequencies due to polarizability. The environment plays a crucial role in each and every case. Again, no unusually high or questionable binding energies are observed for any NO/O_2_ models, as well as in CO. The general trend followed is that positively charged atoms near NO, CO or O_2_ shift the respective stretching vibrational frequencies to higher energies, while negative charges to lower energies. All three gases exhibit a pronounced effect by the environment on their vibrational stretching frequencies, without the need to be bound on a metal (*e.g.*, heme-iron or Cu_B_ site in CcO).

Summarizing, we can accept that the variation of ν(CO) of free CO ligand inside a protein cavity can reach up to 170 cm^−1^ (2,216–2,047 cm^−1^ based on theoretical models presented herein), without excluding more pronounced shifts. Such interactions are of course more complicated and combine effects from hydrogen bonds, positively and negatively charged side chains, but in any case the present study enforces us to reconsider the experimental results on ν(CO) or ν(NO), where bound and unbound states may exert vibrational frequencies in the same region of the spectum.

### 2.2. The Bound Interactions

**Figure 5.**
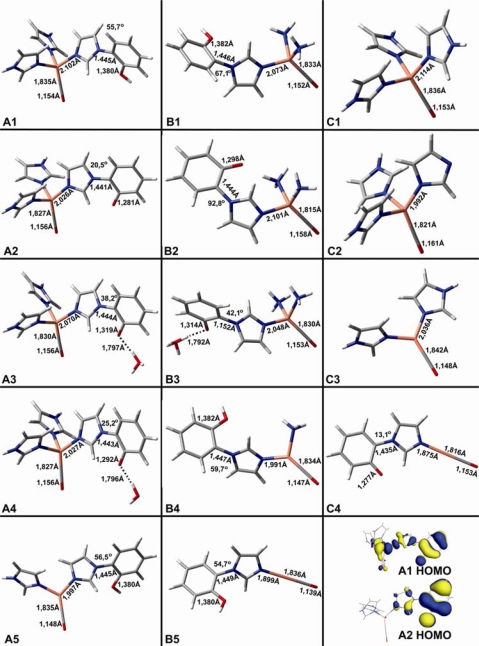
DFT models of diverse Cu-CO complexes in groups A, B and C (figure taken from *J. Phys. Chem. B* **2007**, *111*, 10502–10509, [42]).

**Figure 6.**
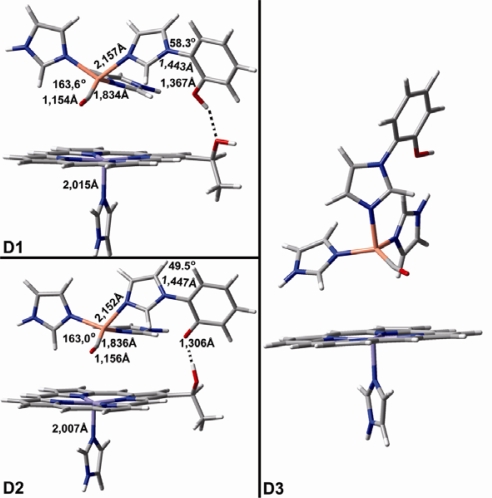
DFT models of diverse heme-iron/Cu_B_-CO complexes of group D (figure taken from *J. Phys. Chem. B* **2007**, *111*, 10502–10509, [42]).

**Cu_B_–CO complexes of Cytochrome *c* Oxidase** [42]. Protonation/deprotonation events of groups near the catalytic center of CcO have been suggested to be involved or not in the delivery of protons for the dioxygen reduction chemistry and proton pumping [48–51]. Thus, it is important to probe such functional properties in the first coordination sphere of Cu_B_. Protonation/deprotonation events at the binuclear center coupled to the dynamics and chemistry occurring at the heme-Fe is crucial in elucidating the functional properties of the enzyme itself. On this line, we have designed four groups of models, referring to them as **A**, **B**, **C** (Figure 5) and **D** (Figure 6). In these models, Cu_B_ (I) is coordinated to three (**A1–A4**), and two (**A5**) imidazoles and the cross-linked His-Tyr is designed as a cross-linked imidazole-phenol (protonated) unit. In **A2–A4** the phenol is deprotonated. In **A3** and **A4** the phenolic O is H-bonded to H_3_O^+^ and H_2_O, respectively. In group **B**, the imidazoles coordinated to Cu_B_ in group **A** are substituted by NH_3_ ligands, while the cross-link Im-phenol unit remains protonated in **B1** and deprotonated in **B2**–**B3**; in **B3** the phenolic O is H-bonded to H_3_O^+^. **B4** is similar to **B1** but with one instead of two coordinated NH_3_ ligands. **B5** lacks both NH_3_ ligands of **B1**. In **C1**–**C2**, Cu_B_ is coordinated to three Imidazoles without the cross linked phenol unit. In **C1**, all imidazoles are protonated and in **C2** one of the three imidazoles is deprotonated. In **C3**, Cu is coordinated to two protonated imidazoles. In **C4**, Cu is coordinated to a deprotonated cross-linked Im-phenol unit. In group **D**, we have optimized structures of the binuclear Cu_B_(I)-Fe(II) center in which a CH_3_CH(OH)-group represents the hydroxyethylgeranylgeranyl or hydroxyethylfarnesyl side chain of the hemes (**D1**–**D2**) or is absent (**D3**). The distance between Cu(I) and Fe(II) is constrained to ~4,4 Å. In Figure 5 the HOMO of **A1** and **A2** are also presented.

Table 1 summarizes the calculated ν(Cu-C), ν(C-O) and selected δ(Cu-C-O) frequencies for the **A-C** models. A close inspection of the data presented in Table 1 shows large frequency shifts in ν(CO) when Cu_B_ looses one of its imidazole (or NH_3_ in simpler models) ligands. When a Cu_B_-Im bond scission occurs, ν(CO) shifts to higher frequency by 43 cm^−1^ (**A5** compared to **A1**), as the C–O bond strengthens due to geometrical and electronic structural changes in Cu. A relatively smaller effect (7 cm^-1^) is calculated for ν(Cu-C) in the same set of models. A change in the protonation state of one of the Cu_B_ imidazole ligands (comparison of **C1** to **C2**) has significant change in the back donation of electron density and therefore the vibrational frequencies with a calculated shift of 20–43 cm^−1^, while deprotonation of the phenol unit (comparison **A1** to **A2**) strengthens the Cu–C and weakens the C–O bond leading to a frequency shift of 11 cm^−1^. In the case where Cu lacks two of the three imidazoles (**C4**) or the two imidazoles are replaced by NH_3_ groups, the deprotonation of the cross-linked phenol unit (**B5**→**C4** or **B1**→**B2**) has a pronounced effect of 120 cm^−1^ and 38 cm^−1^, respectively. The calculated frequencies indicate that electron density due to phenol deprotonation moves mainly to the Cu-C-O entity, altering bond lengths and vibrational frequencies. On the other hand, if Cu is coordinated to two imidazoles (structures **A1**, **A2**), electron density due to phenol deprotonation does not localize to the Cu-C-O moiety, but rather is delocalized on the whole Cu_B_ complex, including the histidine ligands, leading to a reduced Δν(CO) effect of 11 cm^−1^.

Figure 6 shows geometry optimized binuclear models containing both Cu_B_ and heme Fe. Models **A**, **B**, **C** exhibit an almost linear Cu-C-O geometry compared to **D1** and **D2**, where the Cu-C-O tilt and bending is different when the heme Fe is present, but remains unaltered in deprotonation events.

Models **D1** and **D2** were altered in such way that CO is bound to heme-iron rather than to copper (structures not shown). Geometry optimization was performed on the same blyp/dnd (dmol3) level of theory. CO geometry (bending or tilting) is affected as the distance between the metal sites changes. The iron-copper distance was kept constant at 6.0, 5.0 and 4.5 Å for the above models and the rest of the active site was left free to be optimized. The tilt/bend of Fe-C-O moiety was calculated to be 87.9°/178.0° (6.0 Å), 84.7°/173.9° (5.0 Å) and 80.5°/169.1° (4.5 Å). The nature of this distortion either in favor of electrostatic interactions or steric hindrance can lead to discrimination between CO and O_2_ by systems as CcO, Myoglobin (Mb) and Hemoglobin (Hb).

While in dioxygen a favorable overlap between its π*-orbital and Fe-d_z_2 orbital minimizes the binding energy by bending [52], this is not the case for CO, where these orbitals are not close in energy. Thus, the observed M-C-O distortion in CcO is induced. Metal bound CO has a character of M-C^(−)^-O^(+)^. Electrostatic repulsion occurs between the partially positively charged oxygen of CO and the positively charged metal not coordinating CO. Increasing the Fe-Cu_B_ distance (4.5 Å→5.0 Å - step: 0.5/4.5 Å→6.0 Å - step 3 × 0.5) the relative changes in CO distortion (see above) would be proportional to a 1:3 scheme in the case of a steric hindrance-only assumption, as M-C-O would relax to the linear geometry proportionally to the step. In fact, these relative changes in tilt and bend appear to be consistent with a ~1:1.8 scheme. This could be justified by a more complex mechanism including electrostatic interactions in the active site inducing the observed distortions.

As of the importance of electrostatic interactions in M-C-O systems, DFT calculations by Blomberg L. M. *et al*. [1] have shown that Cu_B_ in CcO has a similar effect on the binding energy of CO on Fe as the distal histidine in Mb. In addition, CO binding studies on mutants of Mb by Kozlowski and Spiro [53] have revealed that the steric hindrance by the distal histidine is worth only ca. 1 kcal/mol, while electrostatic interaction by H-bonding is the main reason (85%) [54] for binding CO/O_2_ discrimination.

In the Cu_B_-CO models the changes in the bond lengths of the CO-bound and, thus, the calculated frequency shifts, can be attributed to the increase of electron density on the metal center: as the electron density on the metal centre increases (deprotonation increases the flow of electron density to Cu_B_ as discussed above) more electron density donation to the CO ligand takes place. This increases the M–CO bond strength making it more double-bond-like (M=C=O) which in turn, further weakens the C–O bond by increasing the electron density into the carbonyl antibonding orbitals. Trans-ligands to a carbonyl can have a particularly large effect on the ability of the CO ligand to effectively π-backbond to the metal. For example, two trans π-backbonding ligands will partially compete for the same d-orbital electron density, weakening each others net π-backbonding. Trans-ligand which are a σ-donors can increase the M–CO bond strength (more M=C=O character) by allowing unimpeded metal to CO π-backbonding.

The existence and identity of a reorganization of the Cu_B_ geometry caused by protonation/deprotonation and/or breakage of one of the Cu-N(His) bonds has been a difficult matter to either prove or disprove since Cu_B_ is spectrally silent and therefore no definite spectroscopic evidence had been observed. The ν(CO) frequency of the transient Cu_B_-CO complex determines in great amount the conformational changes (*e.g.,* ligand dissociation) of copper metal site in CcO, while both ν(CO) and ν(Cu-C) are reliable criteria for protonation/deprotonation phenomena. Therefore, we can derive structural information for the Cu_B_-N (His) ligands from the ν(CO) frequency of the Cu_B_^1+^-CO complex. The negative slope in the d(C-O) vs. d(Cu-C) diagram implies the presence of a π-backbonding in the Cu-CO complexes. The calculated ν(CO) frequencies of Cu_B_ under different protonation/deprotontion states and/or ligand-detachement indicate that the ν(CO) frequency dependents strongly on the degree of backbonding. A change in the protonation state of one of the His ligands would have significant changes in the back donation, and thus on the frequency of ν(CO). If one of the His-ligands of Cu_B_ is capable of cycling through the imidazolate, imidazole and imidazolium states then ν(CO) is expected to vary. We have shown [42] that no structural change at Cu_B_ occurs in association with CO binding to and dissociation from heme *a*_3_ in conjunction with the consensus that the pH/pD dependency of the Cu_B_-C-O vibrational frequencies in heme-copper oxidase is not due to deprotonation of the cross-linked tyrosine demonstrates that the environment of Cu_B_ does not serve as a proton-labile site.

**Figure 7.**
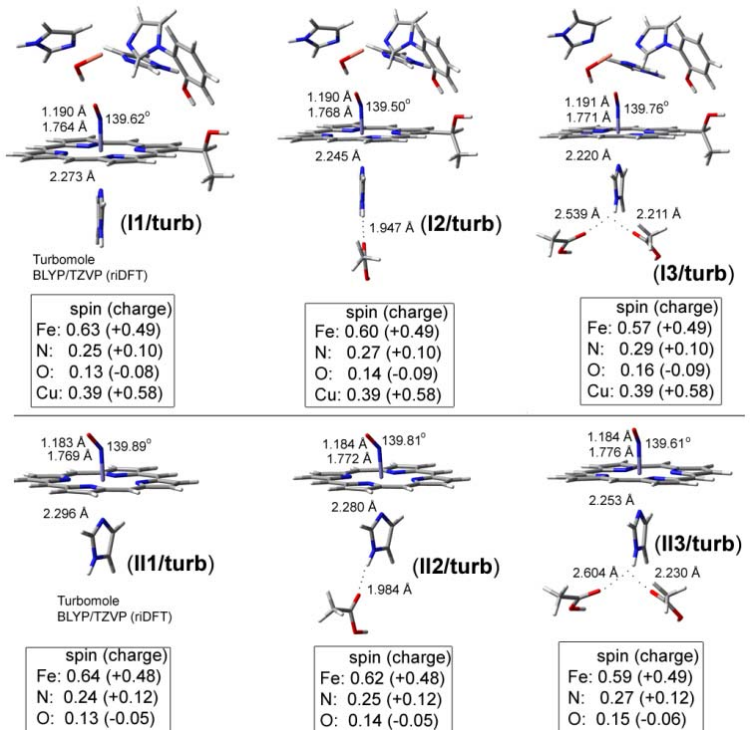
DFT optimized geometries in heme-iron-NO binuclear complexes. Hydrogen bonding network to the proximal area is variable.

**Figure 8.**
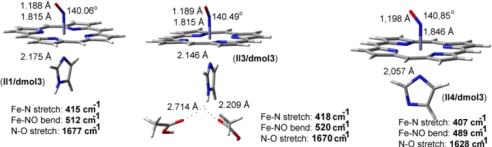
DFT optimized geometries and theoretical vibrational frequencies in heme-iron-NO complexes. Hydrogen bonding network to the proximal area is variable.

**Heme–NO and –CO complexes**. Heme-Fe(II)-NO models are treated as doublet neutral species (without Cu_B_ metal site) or positively charged (+1) with doublet/triplet multiplicity in case of the presence of the Cu_B_ in oxidation states (I) or (II) respectively. In Figure 7 we present DFT models including both Cu_B_(II)-OH and Fe(II)-NO without (**I1**), and with one (**I2**) or two (**I3**) hydrogen bonds to the proximal coordinating Imidazole (a carboxyl acid provides the O-donor atom). Models **II1**, **II2** and **II3** (Figure 8) contain no Cu_B_ site, but exclusively the heme Fe*_a3_*(II) site with different hydrogen bonding networks in the proximal area, without (**II1**), and with one (**II2**) or two (**II3**) carboxyl groups in these networks. Model **II4** (Figure 8) is derived from **II1** by deprotonating the proximal coordinating Imidazole. Figure 7 contains models treated by the ri-blyp/tzvp level of theory by Turbomole software package (highlighted as**/turb**), while Figure 8 by blyp/dnd level of theory in dmol3 module of Materials Studio Suite of programs (highlighted as**/dmol3**). We use diverse software packages in order to evaluate the results in different levels of theory.

**Figure 9.**
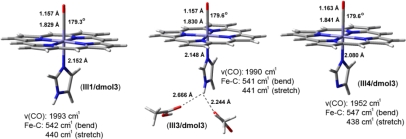
DFT optimized geometries and theoretical vibrational frequencies in heme-iron-CO complexes. Hydrogen bonding network to the proximal area is variable.

In contrast to Fe-NO complexes, heme-CO complexes (Figure 9), where proximal Imidazole is found without (**III1**) and with two (**III3**) hydrogen bonds in the proximal area or with a deprotonated Imidazole (**III4**) the shift in ν(CO) is significant only for the latter case at 40 cm^−1^, in connection to experimental and theoretical studies [40,55].

Theoretical DFT studies on heme-NO complexes of heme proteins conclude that the distal effect is crucial for recognition and discrimination of ligand molecules by the protein [1,56]. As discussed above, it seems that the proximal effect is not significantly higher to be involved in recognition and/or discrination mechanisms by such proteins. We cannot, though, exclude that upon binding, conformational changes are communicated through the proximal site.

To summarize, we conclude that, for CO bound in heme groups, changes to the axial proximal ligand, through either hydrogen bonding networks or deprotonation, do not affect to the same extent, compared to Cu_B_-CO complexes, the Fe-C/C-O bond energies.

## 3. Calculation Methods

**Density Functional Theory on unbound CO, NO and O_2_**. For each structure considered, a full geometry optimization was performed by using the density functional B3LYP method. We used the 6–31 g (d,p) double-*ζ* valence basis set augmented with *p*-PGTOs on H atoms, as implemented in the Gaussian 03 software package [57]. When vibrational frequencies are calculated by electronic structure theory, they can often be improved by scaling, and it is useful to have general scaling factors. Such factors depend on the level of electronic structure theory and the one-electron basis set. It has been established that calculated frequencies may be scaled in various ways [58]. For example, one scaling factor is applied to reproduce the true harmonic frequencies, the true fundamental frequencies, or the zero point energy. To accurately reproduce the experimental ν(CO) of interaction-free gas CO, a scaling factor of ×0.9703 was used for the theoretically calculated ν(CO). A scan for ν(CO) using different basis sets starting from 6–31g (d,p) through 6–311g++(2df, 2pd) showed identical trends (+/−10 cm^−1^) for ν(CO) concerning the environmental effect. Solvation effects count for only a 5 cm^−1^ theoretical shift and, thus, were not taken into account. Scaling factor of ×0.938 on theoretically calculated stretching vibrational ν(O-O) frequencies, to reproduce the experimental value of ν(O-O) in a non-interacting *triplet* O_2_ (1,555 cm^−1^). While, a scaling factor of x0.942 was used for the ν(N-O) vibrational stretching frequencies, based on the ν(N-O) experimental value of NO gas (1,876 cm^−1^). Calculation of the solvent effect (H_2_O) and a basis set scan was also performed for both ν(O-O) and ν(N-O), with negligible variations for ν(X-O) once again (X = N, O).

**Density Functional Theory on bound complexes.** DFT calculations are performed on systems with Cu_B_(I) and heme-Fe(II) leading to a *singlet*, positively charged (+1) mononuclear Cu_B_(I)-CO complex or a binuclear Cu_B_(I)-CO Fe(II) center. For each structure considered, a full geometry optimization was performed using the density functional BLYP method of **dmol3** [59] module in Accelrys Materials Studio 2.21. Double Numerical plus d-functions with a polarization d-function on all non-hydrogen atoms are used for all light elements. For the metals (iron and copper) effective core potential (ECP) from the Stuttgart-Dresden group [60] was used as this is implemented in dmol3 module of Materials Studio (*Accelrys*). The functional GGA/BLYP on a DND basis set used for the calculations is consistent with a BLYP/6–31G(d) level calculation in Gaussian software package. In all cases (convergence tolerance, integration accuracy, SCF tolerance) level fine was selected.

For the **heme–CO or –NO complexes** the ri-blyp functional along with the tzvp triple-*ζ*valence basis set level of theory has been applied, as implemented in the Turbomole software package or the blyp/dnd level of theory when dmol3 module of Materials Studio is mentioned.

A scaling factor has not been applied for the bound model species, as absolute experimental vibrational frequency values depend highly on the system under study and usually refer to either more complicated model-compounds or enzymes. In contrast, all unbound vibrational frequencies have been scaled as small theoretical models in reference (CO, NO and O_2_) have been also studied experimentally as they were designed in theory.

No restrictions in geometry, as well as a Hessian calculation, after the full geometrical optimization of each structure have been applied for all above calculations. Mulliken population analysis was also performed and the results are shown on Figure 7 and 8. All vibrational frequencies stated in this study are harmonic and represent those of the highest intensity.

Unbound (CO, NO and O_2_) and bound (heme-CO, Cu_B_-CO/heme-NO) complexes are treated differently in the level of theory. As the theoretical model used increases in size (unbound to bound states), we have to decrease the level of theory to converge the calculations in a descent amount of cpu-time. This seemed to be a compromise in accuracy and on this line we compared spectroscopic results between b3lyp/lanl2dz based calculations in Gaussian 98 and blyp/dnd based in dmol3 for selected Cu_B_-CO theoretical models in Figure 6. The calculated frequencies (b3lyp) for the Cu-C stretching mode are 380 cm^−1^ for **A1** and 391 cm^−1^ for **A5**. Thus, we observe an A1→A5 + 11 cm^−1^ shift in frequencies in the higher level of theory (b3lyp/lanl2dz) and +7 cm^−1^ for the blyp/dnd (dmol3) level. Both methods calculate a ν(CO) frequency of 2031 cm^−1^ for **A1**, whereas in **A5**, there is a 1 cm^−1^ difference for the ν(CO) frequency. Geometric parameters, such as bond lengths and angles, differ only of a maximum amount of 0.04 Å and 5–6°, respectively, between the two levels of theory. Despite the differences in the absolute values of the vibrational frequencies between the two methods, the calculated frequency shifts follow the same trend. As absolute values usually are of no significant use, trends and vibrational shifts contain important information for experimental spectroscopy.

## 4. Conclusions

Intracavity dynamics play a major role in the function of protein and enzymes [51,61–68]. Ligand molecules are directed into the active site or internal cavities of a protein and/or though channels. The specificity or potency of an enzymatic catalytic reaction relies strongly on the interactions between the protein matrix and the substrate. Density Functional Theory is a potent tool for probing such interactions, even in the cases where a crystal structure has not been solved. Based on the theoretical studies presented in this review, we can conclude that for small molecules like NO and CO, distal interactions become important for ligand discrimination or recognition by the proteins, while proximal effects exert a significantly lower contribution. Nevertheless, we have to observe that in the case of NO, proximal effects, although weak, become more pronounced than in the case of CO. In addition, inner-cavity interactions play a major role in polarizing gases like O_2_, NO and CO. The latter exerts a significantly higher polarizability, as shown by the diversity of ν(CO) vibrational frequencies calculated in models of protein CO-cavity interactions. In the case of CO-bound models of Cu_B_ CcO it seems that deprotonation/protonation or ligand dissociation events in the first coordination sphere of the metal are sensed by the Cu_B_-C-O unit, as they are accompanied by pronounced shifts in both ν(CO) and ν(Cu-C). DFT can be successfully used not only to structurally and spectroscopically probe active sites in proteins, but also cavities. In this way we can link experimental data to an atomistic overview of the systems under study and extract structural information from the experimental data in the most efficient way.

## Figures and Tables

**Table 1.** Theoretical vibrational frequencies in cm^−1^ of the various DFT models in groups A, B and C (table taken from *J. Phys. Chem. B* **2007**, *111*, 10502–10509, [42]).

**Cu_B_-CO models**	**Theoretical vibrations in cm^−1^ (dmol3-BLYP/DND)**		
	**ν(C-O)**	**ν(Cu-C)**	**δ(Cu-C-O**
**A1**	**2031**	**414**	**326**
**A2**	2020	425	
**A3**	2020	420	
**A4**	2023	421	
**A5**	2072	421	
**B1**	**2049**	**419**	
**B2**	2011	442	
**B3**	2040	421	
**B4**	2083	422	
**B5**	2147	444	
**C1**	**2035**	**411**	**324**
**C2**	1992	431	
**C3**	2078	411	
**C4**	2026	468	

**Table 2.** Depicts the vibrational frequencies for **II1**, **II2** and **II3**, without the Cu_B_ site. 7–8 cm^−1^ shifts were calculated in ν(N-O) and ν(Fe-N). For the extreme case of proximal Imidazole deprotonation, a 42 cm^−1^ shift in ν(N-O) is observed.

**Fe-NO models**	**ν(N-O) in cm^−1^**	**ν(Fe-N) in cm^−1^**	**δ(Fe-N-O) in cm^−1^**
**II1/dmol3**	**1677**	**415**	**512**
**II3/dmol3**	1670	418	520
**II4/dmol3**	1628	407	489
